# Supplementary material for: Body mass index is associated with hypoglycaemia in children with acute vomiting and dehydration
Source: PLoS One. 2026 Jan 27;21(1):e0341574. doi: 10.1371/journal.pone.0341574 (PMC12843525; doi:10.1371/journal.pone.0341574)
Supplement: S1 Table — Only original studies including children beyond the neonatal period were included. Studies focusing on hypoglycaemia due to congenital hyperinsulinism, inborn errors of metabolism, fatty acid oxidation defects, glycogen storage diseases, medication-induced hypoglycaemia, or neonatal hypoglycaemia were excluded. Abbreviations: IKH, idiopathic ketotic hypoglycaemia; ED, emergency department; BMI, body mass index; y, years. (DOCX) [file pone.0341574.s001.docx]

**S1 Table. Published original studies reporting clinical factors associated with hypoglycaemia in children: idiopathic ketotic hypoglycaemia versus hypoglycaemia during acute illness.**

| **First author (year)** | **Study type / setting** | **Age range** | **Hypoglycaemia type** | **Associated factors reported** | **Anthropometry analysed** |
| --- | --- | --- | --- | --- | --- |
| **Colle (1964)** | Case series | 1.5–7.5 y | IKH | Young age (but rarely < 1.5 y); prolonged fasting; low body weight | Weight and height; no BMI |
| **Habbick (1971)** | Retrospective case series | 1–6 y | IKH | Young age; fasting; weight <50th percentile | Weight and height; no BMI |
| **Kaplowitz (2019)** | Retrospective cohort | 0.5–6 y | IKH | Age 0.5-6 years; poor oral intake | BMI percentile reported (no association found) |
| **Daly (2003)** | Retrospective ED cohort | 0.6–7 y | Reported as IKH; diagnostic process not described | Young age; male gender; low body weight | Weight only; no BMI |
| **White (2020)** | Retrospective ED cohort | NA | Reported as IKH; diagnostic process not described | Fasting | Not analysed |
| **Bennish (1990)** | Prospective hospital-based study | 2-15 y | Acute illness (gastroenteritis) | Less time with diarrhoea, prolonged fasting | Weight-for-height |
| **Reid (2003)** | Retrospective ED cohort | 0.5-6 y | Acute illness (gastroenteritis) | Female gender, neurolycopenic signs, vomiting more frequent than diarrhoea, home rehydration with water | Not analysed |
| **Reid (2005)** | Retrospective ED cohort | 0.6–4.6 y | Acute illness (gastroenteritis and dehydration) | Duration of vomiting | Not analysed |
| **Qadori (2018)** | Prospective hospital-based study | 1-5 y | Acute illness (gastroenteritis) | Duration of vomiting, age 1-5 years | Not analysed |
| **Ahmad (2024)** | Retrospective hospital-based cohort | Median 3.2 y | Acute illness with acidosis | Vomiting or gastroenteritis; metabolic acidosis | Not analysed |

Only original studies including children beyond the neonatal period were included. Studies focusing on hypoglycaemia due to congenital hyperinsulinism, inborn errors of metabolism, fatty acid oxidation defects, glycogen storage diseases, medication-induced hypoglycaemia, or neonatal hypoglycaemia were excluded. **Abbreviations:** IKH, idiopathic ketotic hypoglycaemia; ED, emergency department; BMI, body mass index; y, years.
