# Supplementary material for: Body mass index is associated with hypoglycaemia in children with acute vomiting and dehydration
Source: PLoS One. 2026 Jan 27;21(1):e0341574. doi: 10.1371/journal.pone.0341574 (PMC12843525; doi:10.1371/journal.pone.0341574)
Supplement: S2 Table — Multivariable logistic regression analyses were performed with hypoglycaemia (≤3.3 mmol/l) as the dependent variable. Covariates included sex, log-transformed BMI (imputed), degree of dehydration, diarrhoea, and oral intake (zero = 1, decreased = 0). Missing BMI values were imputed using multiple imputation by chained equations with predictive mean matching. The imputed datasets were analysed using the same multivariable logistic regression model as in the primary analysis, and regression estimates were pooled across imputations according to Rubin’s rules. Abbreviations: BMI, body mass index. (DOCX) [file pone.0341574.s002.docx]

**S2 Table.** **Multivariable logistic regression analysis of factors associated with hypoglycaemia using multiple imputation for missing BMI data.**

| **Variable** | **OR** | **95 % CI** | **p value** |
| --- | --- | --- | --- |
| Degree of dehydration | 2.430 | 1.654-3.571 | **<0.001** |
| Diarrhoea | 0.307 | 0.161-0.583 | **<0.001** |
| BMI (imputed, log10 transformed) | 0.075 | 0.015-0.391 | **0.002** |
| Zero oral intake | 1.736 | 1.166-2.588 | **0.006** |

Multivariable logistic regression analyses were performed with hypoglycaemia (≤3.3 mmol/l) as the dependent variable. Covariates included sex, log-transformed BMI (imputed), degree of dehydration, diarrhoea, and oral intake (zero = 1, decreased = 0). Missing BMI values were imputed using multiple imputation by chained equations with predictive mean matching. The imputed datasets were analysed using the same multivariable logistic regression model as in the primary analysis, and regression estimates were pooled across imputations according to Rubin’s rules. Abbreviations: BMI, body mass index.
